# The Importance of Shared Decision Making in the Decision to Prevent a Parastomal Hernia With Prosthetic Mesh

**DOI:** 10.3389/jaws.2023.12316

**Published:** 2023-11-10

**Authors:** M. López-Cano, J. M. García-Alamino

**Affiliations:** ^1^ Abdominal Wall Surgery Unit, Department of General Surgery, Hospital Universitari Vall d’Hebron, Universitat Autònoma de Barcelona, Barcelona, Spain; ^2^ Global Health, Gender and Society (GHenderS), Facultat de Ciències de la Salut, Blanquerna-Universitat Ramón Llull, Barcelona, Spain

**Keywords:** decision making, hernia, parastomal hernia, prosthetic mesh, shared decision making (SDM), prevention

In the modern context of surgery, where respect for patient autonomy is paramount, the decision-making process should not be unilateral [1]. In this environment, the Shared Decision Making (SDM) model emerges as an essential tool [2]. This model can be used as a “tool” that allows surgeons to “work” together with their patients in making decisions regarding diagnostic tests, treatments, and/or care plans that involve various interventions [1]. Furthermore, the improved bidirectional communication between surgeon and patient that the practice of SDM entails can limit the use of an intervention that is unlikely to benefit the patient, does not align with their desires, or both [3]. Different ways of carrying out the steps of the SDM process have been described, but essentially, they all describe similar concepts. For its simplicity and clarity, the SHARE Approach developed by the Agency for Healthcare Research and Quality (AHRQ) in the United States can serve as a guide for surgeons less familiar with SDM [4].

Clinical practice guidelines (CPGs) have been defined as “statements that include recommendations intended to optimize patient care that are informed by a systematic review of the evidence and an assessment of the benefits and harms of alternative care options” [5]. Probably, the best current methodology for developing a CPG is the GRADE (Grading of Recommendations Assessment, Development and Evaluation) methodology [6]. However, on many occasions, the recommendations in guidelines are rated as weak in the GRADE system [7–11], indicating the presence of different options or approaches, a delicate balance between risks and benefits, or uncertainty in the evidence. In these circumstances of variability and uncertainty in the recommendation, each patient may make different decisions based on their individual values and preferences, and the “application” of the recommendation should not be a unilateral decision by the surgeon [1]. Therefore, SDM is essential for implementing weak recommendations that are consistent with patients’ values and preferences, both for therapeutic and preventive options [12].

In 2018 guidelines for the prevention and treatment of parastomal hernia (PH) coming from the European Hernia Society (EHS) were published [8]. A recent update of these guidelines (following the GRADE methodology) on the specific topic of using a prosthetic mesh in the prevention of PH has been published [13] and makes a strong recommendation for the use of mesh in cases of patients at high risk of PH after end colostomy construction (patients with a history of abdominal wall hernia, connective tissue disorder, obesity, or undergoing chemotherapy) and a life expectancy of over 2 years. However, when it comes to generalizing to other types of patients (no high risk), they conditionally (weak) suggest/recommend the use of a non-absorbable synthetic prophylactic mesh in end colostomy construction [13].

In the context described above, the use of SDM appears indispensable for a significant majority of patients when deciding whether to implant a mesh to reduce the risk of PH. Some of the reasons why SDM is crucial in this decision include:

*Individual values and preferences:* Each patient has unique values, goals, and expectations. Considering their own experience and unique situation, patients can make better-informed decisions when they understand the information provided and how it relates to their current clinical context and possible future outcomes. Some may prioritize preventing a hernia, given its potential impact on quality of life, while others may be more cautious about introducing synthetic materials into their bodies.

*Clinical outcomes and quality of life:* Medical decisions not only affect objective clinical outcomes but also the patient’s perception of their quality of life. Shared decision-making allows for a more comprehensive assessment of how an intervention might affect daily life, emotional wellbeing, and physical activities. This is particularly relevant when there is a lack of reported patient evidence on their health outcomes (e.g., patient-reported outcomes).

*Promoting patient autonomy:* SDM strengthens patient autonomy by making them active participants in the decision-making process. This not only aligns with ethical principles in medicine but can also increase patient satisfaction and adherence to postoperative recommendations.

*Education and understanding:* This model promotes a bidirectional exchange of information. While the physician provides evidence-based information, the patient contributes their personal knowledge and unique context. This ensures that the patient has a comprehensive understanding of the intervention and its potential implications.

*Reducing post-decision regrets:* Making medical decisions can be stressful, and decisions made without sufficient deliberation or understanding can lead to regrets. SDM reduces the likelihood that patients will feel dissatisfied or uncertain about their long-term decisions.

In conclusion, the decision to reduce the risk of a PH with prosthetic mesh should not be taken lightly. It is a choice that has significant implications for health outcomes and quality of life, with recent literature questioning the effectiveness of non-absorbable synthetic mesh in preventing PH in the context of an end colostomy when long-term follow-up is considered [14]. The SDM model emerges as an invaluable tool to ensure that an informed, balanced decision is made, aligned with the individual values and desires of the patient.
